# Risperidone Pellets, Pycnogenol^®^, and Glucomannan Gummy Formulation for Managing Weight Gain and ADHD in Autistic Children

**DOI:** 10.3390/pharmaceutics16081062

**Published:** 2024-08-13

**Authors:** Rawand M. Daghmash, Mai S. Khanfar, Ruba S. Darweesh

**Affiliations:** Department of Pharmaceutical Technology, Faculty of Pharmacy, Jordan University of Science and Technology, P.O. Box 3030, Irbid 22110, Jordan; rmdaghmash19@ph.just.edu.jo (R.M.D.); rsdarweesh@just.edu.jo (R.S.D.)

**Keywords:** autism, risperidone, pellets, medicated gummies, glucomannan, Pycnogenol

## Abstract

Gummy formulations are defined as gradually or slowly released solid oral dosage forms. Risperidone is an atypical antipsychotic medication used to treat schizophrenia and autism-related irritability. This study presents the development of visually appealing, patient-tailored medicated gummies that act as a novel pharmaceutical form of Risperidone for pediatrics. In this study, two gummy bases were used, one containing Glucomannan and the other containing Gelatin as a gelling agent, where these gummy bases were loaded with coated Risperidone pellets with a controlled release layer. The final products were evaluated for their pH, viscosity, content uniformity, drug content, and dissolution profile. Both formulas showed proper rheology and met content and weight uniformity standards. The release rates for F1 and F2 in the acidic media were 25% and 11%, respectively, after 2 h. At the same time, a full-release profile for Risperidone was noticed in both formulae at pH 6.8 where the release lasts for 24 h. It can be concluded that the chewable semi-solid dosages (gummies) filled with coated pellets are suitable for pediatric patients since pediatrics have drug-related problems which can be solved using high gastro-resistance coated pellets, which also shows a proper release profile for the drug.

## 1. Introduction

Attention-deficit/hyperactivity disorder (ADHD) and Autism spectrum disorder (ASD) are both considered as chronic neurodevelopmental disorders that might frequently occur with other psychiatric conditions [1]. In population- and community-based investigations, the average incidence of ADHD symptoms in people with ASD ranges between 13 and 50% [2,3,4]. Furthermore, patients diagnosed with ASD and ADHD have symptoms that are more severely incapacitating on multiple levels, which result in deficits in behavioral, adaptive ability, and executive control than patients with just a single diagnosis [5].

The only atypical antipsychotic medications that the US FDA approved for treating irritability and related aggressive behaviors in ASD patients are Risperidone and Aripiprazole [6,7,8]. Additionally, these medications appear to have an effect in lowering both hyperactivity and stereotypy in patients diagnosed with ASD [6]. Mainly, Risperidone is frequently prescribed to comorbid ASD and ADHD patients, according to studies [7,8].

Risperidone mainly blocks the postsynaptic serotonin receptors [9,10]. It is mainly used for the management of schizophrenia (acute and chronic) and irritability associated with autism with severe behavioral issues [10]. Risperidone, is FDA approved to be used in 5–16 years aged children to treat irritability associated with autism [11].

Risperidone has many side effects such as, weight gain, dyslipidemia, insulin resistance, and hyperglycemia [12,13,14]. It is well-known how these metabolic disorders affect morbidity and mortality [15]. In order to manage weight gain and ADHD symptoms, a combination therapy between Risperidone and two other plant extracts might be helpful in resolving these issues.

According to several reports, Pycnogenol^®^ has a positive impact on ADHD patients. According to the monograph “Maritime Pine Extract of the US Pharmacopeia”, Pycnogenol^®^ is a unique extract obtained from the barks of French maritime pine (Pinup pinaster) [16]. This extract is a blend of catechin and taxifolin (procyanidins) as a major constituent, about 85%, and gallic, caffeic, and ferulic acid (phenolic acids), which are considered a minor constituent, each one of them has its own biological and clinical effects [16,17]. Studies have shown that giving Pycnogenol^®^ to children diagnosed with ADHD reduced ADHD children’s hyperactivity and improved their attention, visual–motor coordination, and concentration significantly [18].

Furthermore, one of the biggest threats to global health is obesity, and ongoing research is being conducted to find a solution that will effectively contribute to the reduction of body mass [19]. The most recent weight management guidelines offer all-encompassing lifestyle interventions, such as counseling and education, to shed pounds. However, patients need help adhering to these behavioral changes in clinical practice. The lack of an adequate standard treatment option for children who are overweight or obese motivates research into supportive modalities [20].

To solve the weight gain problem associated with the usage of Risperidone, a water-soluble dietary fiber, Glucomannan extracted from Amorphophallus konjac, can be used to manage obesity and weight gain problems. Glucomannan is marketed for weight loss in many nations. The precise mechanisms by which Glucomannan might act are unknown. Glucomannan has been found to be able to form a highly viscous gel with a significant volume, which slows gastric emptying by heightening the feeling of satiety [21,22].

Oral route administration is favored over other routes because medications can be administered by the patient and are more acceptable to them [23]. Nowadays, an innovative drug delivery system is used in order to treat local and systemic issues; researchers focus on developing medicated gummies [24]. At the same time, other dosage forms are needed for patients who cannot take medications in the form of tablets or capsules, which has encouraged compounding pharmacies to make different choices [25,26,27].

Chewable gummies come within the lozenge family dosage forms. Lozenges are considered as a solid or semi-solid dosage form which may dissolve, disintegrate, or be chewed in the mouth. Lozenges are used for both local and systemic drug delivery. There are three different types of lozenges: hard, soft, and chewy. According to reports, gelatin gummy candies and other soft chewable dose forms may be simpler, appealing, and more natural for children to chew than chewable pills [26,28,29].

Gummies, small colorful and gummy candy, have grabbed the hearts of millions of people all over the world. These snacks, with their chewy texture and variety of tastes, are not only a treat for the taste senses, but also come in novel nutritional supplement or medicated forms. Gummies provide a distinct sensory experience that extends far beyond their plain sweet flavor. They come in a wide range of tastes, from the most classic to the most unusual, and offer consumers a wonderful taste experience. This variety extends beyond flavors; the forms and colors of gummies are equally diverse, making them ideal for both children and adults [30].

One of the primary advantages associated with gummies is their simplicity of intake. Many people find taking tablets or capsules unpleasant or challenging. Gummies, with their smooth texture and pleasant taste, are an appealing option. This feature is especially appreciated by children, the elderly, and others who have trouble swallowing solid pills, since gummies are chewable, easy to swallow, tasty, lightweight, and easy to digest. Additionally, gummies are convenient for on-the-go eating. They do not require water or extra preparation, making them ideal for persons who lead active lifestyles or travel frequently. This ease guarantees that required medications are taken on a regular basis, which is critical to their efficiency. The convenience of administration of gummies also improves compliance. Consumption of medication becomes an enjoyable process rather than a task, people are more likely to stick to a regular pattern, and consumers find it simpler to take their treatment, increasing the potential health advantages [29,31,32,33].

On the other hand, the disadvantages associated with gummies are mainly the overconsumption of the gummies, due to their attractive appearance and delightful taste. The risk is particularly greater for children, who may be unable to distinguish between ordinary and medicated gummies. It is therefore critical for parents and guardians to keep track of their young children’s consumption of gummies and educate them on the nature and appropriate usage of such products. The basic weakness of the gummies is a lack of room for the “active ingredients”. Indeed, a 2 g capsule can hold 2 g of active substances, but a 2 g gummy can only hold 200 or 300 mg of active components. This is owing to the importance of the gelling solution (most often fruit pectin) and sweetening solution (based on sugar or sweeteners for sugar-free variants), which are required to create the sweet flavor that makes gummies such a pleasurable product to ingest. But small dosed active ingredient can be easily integrated to the gummy base [30].

Gummy permits the active ingredients (APIs) to either become dispersed or dissolved in a gel matrix, which is considered as soft, pliable, and elastic [34]. This gel matrix requires a slight force to chew the formulation, permitting the ease of water penetration into the gel structure and initiating salivary API solubilization. The gummy contains mainly flavoring agents, sweeteners, water, sugar, and an ingredient that forms gels. Sweeteners and flavors are also used to boost customer acceptance and mask the bitterness of API [31]. A gelling agent that creates a matrix structure is crucial in gummy preparations. The preparation’s physical properties, particularly its rheology and texture, are greatly influenced by the nature and amount of the gelling agent [35]. Animal bones can be used to extract the protein gelatin. Gelatin’s physical characteristics make it chewier and reversible (it can change from a gel to a liquid when heated and back again when cooled), melt in the mouth, and produce thermos-reversible gels. Gelatin is added during production to improve the suppleness of jelly sweets made with agar [36].

Apart from the known safety issues brought on by the fact that the gelatin used comes from the connective tissue of animals, primarily cows, it has no inherent nutritional or health advantages. Therefore, it would be desirable to have gummy candies in which soluble fibers of plant origin entirely or partially replace gelatin, provided that this substitution has no adverse effects on the organoleptic properties of the candy [29]. So, soluble dietary fibers like inulin, Glucomannan, and partially hydrolyzed guar gum can all be used effectively. Particularly preferred is Glucomannan [37]. As in our research, the medicated gummies can also serve as a carrier or delivery system for APIs. Vegetable fibers found in gummy candies make them advantageous because they allow up to 5 g of dietary fiber to be packed into a minimal volume, guaranteeing that one of these candies, consumed daily, will satisfy the body’s need for dietary fiber and regularize intestinal function, acting on pain and abdominal swelling, constipation, and diarrhea. Even pediatric patients, for whom conventional fiber intake frequently presents acceptance problems, find the administration method according to the invention to have a delightful flavor [38].

This study aims to formulate previously prepared coated Risperidone pellets combined with Pycnogenol^®^ and Glucomannan as a gummy using two different gelling agents.

## 2. Materials and Methods

### 2.1. Materials

Risperidone pure standard (Jubilant Pharmova, Noida, Uttar Pradesh, India) was kindly donated from Tabuk Pharmaceuticals Co., Amman, Jordan. Soluplus^®^ (O.BASF the Chemical Company, Ludwigshafen, Germany); Hydroxypropylmethylcellulose (HPMC) or Methocel^®^ K4M (Only Chemical, Shanghai, China) was applied as a binder and sub-coating film layer; Talc (Charles, B. Chrystal Company, Inc., New York, NY, USA); nonpareil seeds 18/20, 50/35 and 30/35 mesh was a kind donation from (pharma-a-spheresTM, Tornesch, Germany); Eudragit RS 100^®^ (Evonik Industries AG, Essen, Germany) was used as a controlled release layer; Triethyl citrate (Sigma-Aldrich^®^, Darmstadt, Germany); Hydrochloric acid (CARL OERBA Reagents, Milano, Italy); Sodium hydroxide (Thermo Fisher Scientific, Wushan, Germany); Potassium chloride (Thermo Fisher Scientific, Wushan, Germany); Potassium dihydrogen phosphate (AZ chem, Gezina, Spain); Methanol (CARL OERBA Reagents, Milano, Italy); Acetone (ARPADIS, Düsseldorf, Germany); Acetonitrile HPLC grade (Thermo Fisher Scientific, Wushan, Germany); Trifluoroacetic acid (Merck, Darmstadt, Germany); Ammonium hydroxide (ALPHACHEM, Mississauga, ON, Canada); Disodium hydrogen phosphate (Thermo Fisher Scientific, Wushan, Germany); Sucrose is used as a sweetener (CARGILL, Minnetonka, MN, USA); Glucose syrup is used as a flavor enhancer (CARGILL, Minneapolis, MN, USA); Starch used to maintain hardness and chewiness (CARGILL, Minneapolis, MN, USA); Citric acid work as a taste enhancer (Chemtex, West Bengal, India); Flavoring agent (strawberry concentrate) (Foster Clarck’s, Estate San Gwann, Malta); Food coloring (Red dye) (Foster Clarck’s, Estate San Gwann, Malta); Glucomannan used as an alternative to gelatin (NOW FOODS, Los Angeles, CA, USA); Gelatin (Merck KGaA, Darmstadt, Germany); Potassium sorbate used as a preservative (FoodChem, Los Angeles, CA, USA).

### 2.2. Methods

#### 2.2.1. Preparation Risperidone Nanosuspension

A Risperidone nanosuspension was prepared using a method optimized for efficiency, with detailed procedures to be disclosed in a forthcoming publication. Initially, 500 mg of Risperidone was dissolved in 50 mL of Acetone (organic phase), followed by stirring at 400 RPM and subsequent sonication for 15 min utilizing an Ultrasonic Processor Model UP400S (Hielscher, Teltow, Germany). Simultaneously, 1250 mg of Soluplus^®^ was dissolved in 500 mL of Deionized Water (DW) (aqueous phase) and stirred at 1000 RPM until completely dissolved. The organic phase was then introduced drop by drop into the aqueous phase by using a programmed syringe pump Model SP-300 (Next Advance, New York, NY, USA) in order to introduce the organic phase at a rate of 0.5 mL/min under magnetic stirring at 1000 RPM. The temperature was set for 2 h. at 60 °C. Finally, stirring speed was lowered to 250 RPM, and the mixture was left at room temperature overnight until complete evaporation of the organic phase. This process must be repeated several times in order to obtain a final formula containing 7.5 g Risperidone.

#### 2.2.2. Preparation of Coated Risperidone Pellets

The previously prepared Risperidone nanosuspension which contained 7.5 g of Risperidone and 18.75 g of Soluplus^®^ was mixed with 100.05 g of HPMC K4M used as a binder and 202.5 g of talc used as an anti-tacking agent onto, and then sprayed at 1500 g of nonpareil seeds 35/30 mesh using the fluid-bed granulator process using UniGlatt fluid bed Granulator/Coater (Glatt^®^ GmbH Systemtechnik, Binzen, Germany) to obtain Risperidone pellets using the parameters shown in Table 1. Risperidone nanosuspension/HPMC K4M mixture must be maintained under agitation during the whole process at 1000 RPM.

In order to obtain Risperidone-coated pellets, 20% Eudragit RS 100^®^ was used to obtain controlled-release pellets. So, for each 1500 g pellet we need to add 1500 g of Ethanol (96%), 300 g of Eudragit RS 100^®^, 300 g of talc, and 30 g of triethyl citrate (TEC), mainly used as a plasticizer. Reverse osmosis (RO) water was also added, and its quantity was the same as Ethanol in order to dilute Ethanol.

At first, 1500 mL of absolute Ethanol was divided into portions where the first portion was used to dissolve 20% Eudragit RS 100^®^ (300 g) and stirred at 700 RPM. Later, the same amount of RO water was added until a clear solution was obtained. The second portion of Ethanol was used to dissolve 30 g TEC and 300 g talc using a homogenizer at 4000 RPM for two minutes. After that, the second solution was added to the first solution at a continuous stirring under 700 RPM. After that, the rest of the RO water quantity was added and stirred until a homogenous dispersion was obtained. This dispersion was sprayed using UniGlatt fluid bed Granulator/Coater (Glatt^®^ GmbH Systemtechnik, Binzen, Germany) at the previously prepared Risperidone Nano suspension/HPMC K4M Pellet.

#### 2.2.3. Preparation of the Gummy Base

A Sucrose mixture (A) and Gelatin or Glucomannan solution (B) were prepared to make gummy formulas. In order to prepare the Sucrose mixture (A), 30% sucrose, 60% glucose D60, and 10% water were mixed, heated to 120 °C, and then cooled to 65–75 °C, and kept under slow stirring speed.

Gelatin or Glucomannan Solution (B) was prepared by mixing 30% Glucomannan (F1) or Gelatin (F2) with 70% water. The mixture was then heated to 50°C and stirred until a homogeneous mixture appeared.

Finally, 80% of mixture A and 20% of mixture B were mixed; then, flavoring agents (fruit concentrate), dyes, citric acid, 25% of sucrose quantity, 8 mL of potassium sorbate solution (1:3 potassium sorbate: water) were added. The ingredients were mixed slowly at 50 °C until a homogeneous mixture was obtained [39]. The last step was pouring the resultant mixture into molds sprinkled with cornstarch. After that, the molds were left overnight at 18 °C. Finally, the prepared gummy candies were removed from the molds. The prepared gummy ingredients are shown in Table 2.

#### 2.2.4. Preparation of Medicated Gummy

A mixture of coated-Risperidone pellets (containing 1 mg Risperidone), 40 mg Pycnogenol^®^, and 1 gm Glucomannan were mixed and dispersed in a small amount of melted base equivalent to the mold capacity at 25 °C. Subsequently the whole mixture was molded and left overnight at 18 °C. It was then stored at 4 °C in the fridge until use.

For viscosity (thickness) and pH level determination, allow 10 min for the medicated gummy to melt. The final produced products was a heart-shaped (length 1.5 cm, width 1.8 cm, and thickness 0.8 cm), disc- (diameter 1.8 cm, thickness 0.8 cm), or bear-shaped (length 1.8 cm, width 1 cm, and thickness 0.8 cm) red gummy.

#### 2.2.5. Packaging

Each gummy was wrapped individually in foiled paper and kept in a tightly closed, light-resistant container.

Labeling: “Use only as directed, store in the refrigerator, and must be chewed before swallowing”.

Storage: The medication was stored in the refrigerator.

#### 2.2.6. Gummy Candies Evaluation

##### pH Level Determination

The pH meter Model Thermo Orion 320 PerpHect LogR Meter Basic Benchtop (Thermo Scientific Orion, MA, USA) was used to determine the final product pH value. This was accomplished by dipping the pH meter in a melted pre-weighed 1 g medicated gummies for either F1 or F2 after that, they were diluted by adding 20 mL water. And because the product was acidic, the pH was used to ensure its storage durability [40].

##### Viscosity Test (Thickness)

A viscosity test was conducted by Brookfield METEK DV3T viscometer (Brookfield Engineering, MA, USA) using plate number 1 to evaluate the viscosity of both F1 and F2. Viscosity measures a liquid’s ability to flow in a unit of time, and the higher the viscosity value indicates the more excellent resistance or difficulty in flowing [41]. Theoretically, F1 and F2 can have different viscosities depending on the used gelling agent (Glucomannan or gelatin).

##### Determination of Visual Appearance and Weight Uniformity

The gummies’ visual appearance and weight uniformity were examined to evaluate the organoleptic properties, verify accuracy, and ensure design reproducibility. Twenty gummies from each mold models (disc-, heart-shaped, and bear-shaped) for either F1 and F2 were examined using the technical procedures of the US Pharmacopoeia [42]. Electronic balance Model PW124 Lab Balance (Ae Adam, Oxford, MI, USA) was used to weigh each medicated gummy separately to assess the gummies’ weight homogeneity. The average weight was calculated and depending on the US Pharmacopoeia, each weight variation must not show a difference of more than 5% from the average weight (weight compliance limitations) [42].

##### Determination of Risperidone Content

To determine whether the individual Risperidone contents in the medicated gummy were within the US Pharmacopoeia specification, where the content must be between 85% and 115% of the average content. In order to determine Risperidone content, USP monograph was used [43,44]. Since chewable gummies do not require a particular test, a tablet-appropriate approach was selected; according to this guideline, if Risperidone content in medicated gummy falls between 85% and 115% of the average content, then the medicated gummies passes the test [44]. The test was done in triplicates. Drug content was determined using High-Performance Liquid Chromatography (HPLC) (SHIMADZU, Kyoto, Japan) supplied with an autoinjector and UV detector (SHIMADZU, Kyoto, Japan) using the USP method for Risperidone. The apparatus used, the reagents needed, and the procedure are stated in Table 3 [43].

Preparation of Standard Solution

In order to obtain a standard solution, 48 mg of Risperidone added to 200 mL volumetric flask, then dissolved by the diluent then sonicated for 10 min. The volume was then completed using the same diluent and mixed well. After that, 5 mL from the previous solution were transferred to 200 mL diluent, then mixed well and filtrated using 45 µm nylon filters. Serial dilutions were made from the prepared standard solution.

Preparation Sample Solution

A single medicated gummy containing 0.295 gm pellets (1 mg Risperidone) was placed in 50 mL volumetric flask. After that, it was diluted using the diluent, then sonicated for 60 min. The volume was completed with the diluent and centrifuged for 5 min at 4500 rpm. Finally, the resultant solution was filtrated using 45 µm nylon filters.

##### In Vitro Drug Release

Dissolution for the produced gummy formulas F1 and F2 was done using USP Dissolution apparatus type II, Model RC-8DS (Tianjin Guoming, Tianjin Shi, China), and samples were analyzed using High-Performance Liquid Chromatography (HPLC) (SHIMADZU; Kyoto, Japan) supplied with an autoinjector and UV detector (SHIMADZU; Kyoto, Japan), using the USP method for Risperidone as shown in Table 4.

Dissolution studies were conducted for F1, F1 crushed, F2, and F2 crushed, also coated pellets formula containing 1 mg of Risperidone, and non-coated pellets formula containing 1 mg of Risperidone. Each one of them were placed in six dissolution vessels where each one filled with 500 mL dissolution medium equilibrated at 37.0 ± 0.5 °C and operated as stated in Table 4. 10 mL samples were withdrawn and filtrated on a specific time schedule and the amount withdrawn was directly replaced by the same amount of a freshly prepared buffer.

##### Gummies Stability Studies

Due to the soft texture of the produced gummies, it must be stored in the refrigerator to maintain its hardness, so according to ICH recommendations (1995) for the refrigerated dosage forms, the stability performance of gummies must be evaluated at 5 ± 3 °C for 12 months for long-term stability testing [45]. In this study, the formulas were placed in the fridge at 5 ± 3 °C for six months only. The analysis for the samples was performed after the 1st, the 3rd, and the 6th months of storage, where viscosity, pH, organoleptic characteristics, assay, and in vitro dissolution for F1 and F2 were done.

#### 2.2.7. Drug Characterization

##### Differential Scanning Calorimetry (DSC)

Using DSC Model Phoenix DSC 204 F1 (Netzsch, Waldkraiburg, Germany) Samples of non-formulated Risperidone, coated Risperidone pellets, Physical mix (PM), Glucomannan gummy base, Gelatin gummy base, and the produced medicated gummies (F1 and F2) were analyzed. Nitrogen gas flow rate was maintained at 50 mL/min all the samples were placed on a sample aluminum pan after that they get heated from 0 to 300 °C at a rate of 5 °C/min, then the thermograms were detected.

##### X-ray Diffraction (XRD)

Using X-ray powder diffractometer (Ultima IV X-ray diffractometer, Rigaku, Japan), samples of non-formulated Risperidone, coated Risperidone pellets, PM, Glucomannan gummy base, Gelatin gummy base, and the produced medicated gummies (F1 and F2) were analyzed. Samples of non-formulated Risperidone, coated Risperidone pellets, PM were grinded or crushed into a fine powder material using mortar and pestle and remixed to assure sample homogeneity. While Glucomannan gummy base, Gelatin gummy base, and F1 and F2 Samples were lyophilized for 24 h. to get rid of moisture then grinded. After that, the powder samples were over filled in the sample holder plate, then pressed using the pressing block to obtain a smooth surface, after that the extra powder removed. All the samples analyzed using cobalt radiation at 40 kV and a current of 40 mA. The diffraction angles (2θ) ranged from 3° to 60°. The step scan mode was used with a step size of 0.02° and a speed of 3.0 degree/min [46].

#### 2.2.8. Statistical Analysis

JMP (pro 17, SAS Institute Inc., Cary, NC, USA, 1989–2023) was used for statistical analysis. So, One-way ANOVA was employed to discriminate the produced formulas’ characteristics. These tests were done to understand the influence of Glucomannan and gelatin in the gummy base on the medicated gummies’ pH, viscosity, weight uniformity, and texture. The results were considered significant when the *p*-value < 0.05.

## 3. Results

### 3.1. Gummy Candies Evaluation

#### 3.1.1. pH Level Determination

Results shown in Table 5 revealed that F1 and F2 had pH values of 3.46 and 3.42, respectively. These values were examined using JMP (pro 17) software. The normality and homogeneity tests found the data to be homogeneous and normally distributed (* *p*-value > 0.05). Additionally, there was no significant difference between the F1 and F2 as assured by using *t*-test, where the test *p*-value was * 0.027 > 0.05. Knowing that the ideal pH ranges for gummy formation must be acidic near the pH of 3.2 [47], it is necessary to measure the pH value precisely, in the presence of the active materials used in the preparation of the gummy base such as potassium sorbate and citric acid. From the results, it was found that the produced formulas are acidic and meet the specification regarding the pH.

#### 3.1.2. Viscosity Test (Thickness)

**Figure 1:**
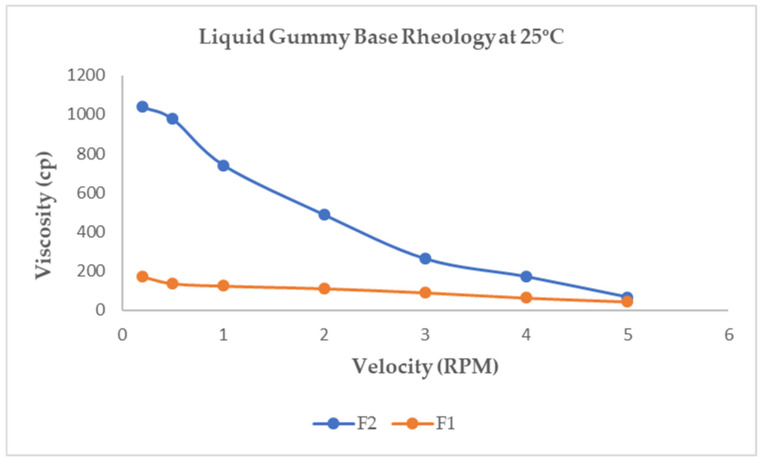
The viscosity profile for F1 and F2.

Viscosity refers to the amount of fluid that flows in a given amount of time. The substance is more complex or resistant to flowing when viscosity increases. ANOVA analysis was not necessary because formulations F1 and F2 were found to be highly significantly different based on the *t*-test results. The outcomes for a liquid gummy base are displayed in Figure 1. Findings were obtained using varying speeds. F1 and F2 showed a pseudo-plastic or shear-thinning behavior, but F2 was extremely pseudo-plastic [48]. The findings demonstrated a negative correlation between viscosity and shear rate, indicative of a non-Newtonian pseudo-plastic flow characteristic (shear-thinning), as seen in Figure 1.

The non-Newtonian’s viscosity decreased with an increased sharing rate [49]. So, F2-containing Gelatin had a higher viscosity than F1-containing Glucomannan. F2 viscosity ranges from 67.44 ± 9.81 cps to 1040 ± 34.6 cps, while F1 viscosity ranges from 44.7 ± 6.22 cps to 170 ± 11.05 cps.

#### 3.1.3. Test for Weight Uniformity and Visual Analysis

One of the things that must be taken in to account during the production process is weight uniformity. The weight differences between each gummy and the total weight were measured in order to determine the weight uniformity. All of the produced gummies (circular, heart shape, bear-shaped) weights were measured to determine the upper- and lower weight limits according to the standard. As stated in the USP, the weight difference must not exceed 5% deviated from the average weight [42,50]. The results showed that all of the produced gummy weights met the acceptance criteria as shown in Table 6, where the gummy weights were found to be consistent irrespective of the mold type used or formula employed.

**Figure 2:**
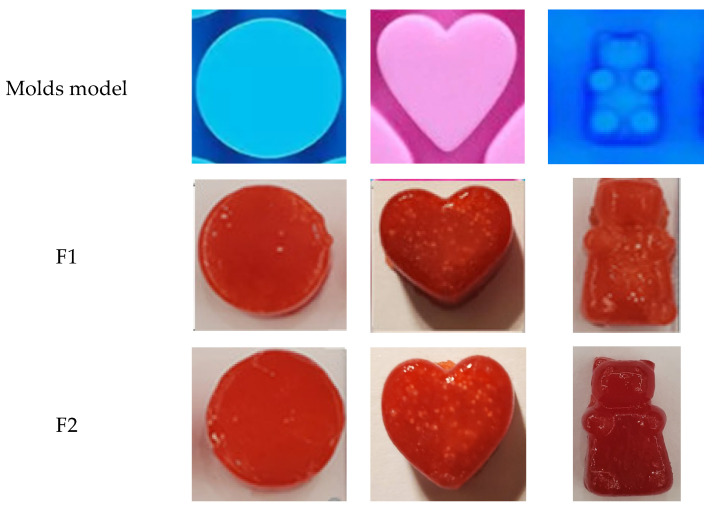
The three molds used and the produced Medicated Gummies formulations.

In keeping with this, it was shown that these medicated gummies were effectively made to satisfy patient dosage needs and that the adaptability of the design can enhance medicine acceptability and treatment compliance. Figure 2 shows the three produced shapes from F1 and F2.

The pellets were easily visible in the produced medicated gummies, confirming their physical integrity throughout the manufacturing process, and verifying the applicability of both formulas. Every medicated gummy had a delicious appearance, a shiny color, and a pleasant smell—qualities necessary when catering to specific picky population segments, including children. Additionally, using pellets provides an additional way to hide the flavor of the active substances.

#### 3.1.4. Determination of Risperidone Content

During F1 and F2 development, coated Risperidone pellets were mixed immediately with Glucomannan and Pycnogenol^®^ and subsequently added to the entire melted gummy matrix. Even though the composition of the gummy base varied significantly, Risperidone was effectively assessed in both formulations. As a result, Risperidone content met the USP specification [43,44]. The drug content values for were found to be 102.4 ± 4.23% for F1, and 99.96 ± 3.58% for F2.

#### 3.1.5. In Vitro Drug Release

**Figure 3:**
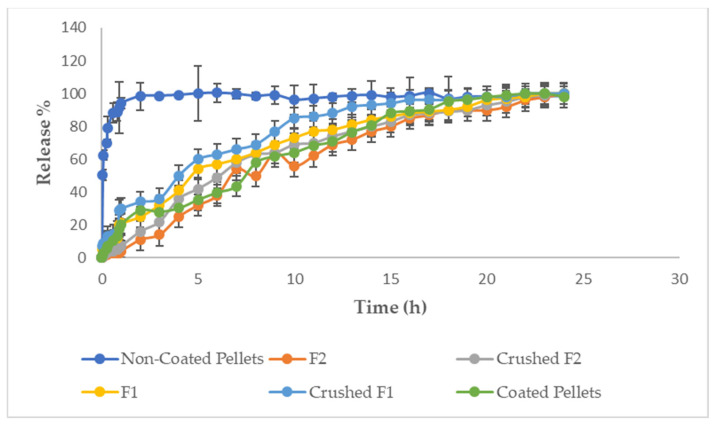
Release time profile for F1, F1 crushed, F2, F2 crushed, coated pellets, and non-coated Risperidone pellets.

As shown in Figure 3, the dissolution tests showed that F1 gave a 25% API release after 2 h in the acidic media, while the F1 crushed showed a release of 34% with a 9% difference from non-crushed formula. So, F1 showed a lower release rate than F1 crushed. In the case of F2, the release rate after 2 h in the acidic medium was found to be 11%, while in case of F2 crushed it showed a release of 16% with a 5% difference from the non-crushed formula. And the difference in the release between F1 and F2 was due to the difference in the viscosity between Glucomannan and Gelatin. Therefore, Gelatin used in F2 as a gummy base gave a slower release profile than Glucomannan used in F1 as a gummy base, and that is due to Gelatin higher viscosity values when compared to Glucomannan. Moreover, by comparing gummies formulation to the coated Risperidone Pellets formulation and the uncoated pellets that were prepared in our previous research, the release of the coated Risperidone Pellets after 2 h in the acidic media was 29%, while the uncoated Risperidone pellets gave 85.26% (burst release). So, both F1 and F2 produced satisfactory release profiles in the acidic media.

A full release of Risperidone was noticed in case of F1, F1 crushed, F2, and F2 crushed up to 100% and last for 24 h., and in case of coated Risperidone pellets the release was complete and lasts for 22 h. This difference in the time where the release ends, due to the presence of Gelatin and Glucomannan in the gummy base affects the release of the drug. Meanwhile, in the case of Gelatin, there is a complex molecular chain entanglement, forming a compacted network structure with a more robust capacity for water-holding [51]. When Gelatin is placed in aqueous media, it swells due to hydration. Sugars and polyols in the formula had an essential role in stabilizing the Gelatin gel and the Gelatin networks in water; by this, the Gelatin gel rigidity will be enhanced [52]. So, a lower and slower solvent penetration through the Gelatin structure leads to a slower drug release from the formula [53]. Also, Glucomannan had an effect on preventing the fast or burst release of drugs [51]. Glucomannan is known as a dietary fiber considered highly absorbent and water-soluble, with the highest viscosity when compared to the other dietary fibers. Under alkaline conditions, Glucomannan creates an unusual, resilient, elastic, and heat-stable gel layer. Furthermore, Glucomannan can regulate the release of drugs [54]. So, both Glucomannan and Gelatin gummy base gelling agents had an influence on the drug release profile, by preventing the burst release and enabling a more controlled or extended drug release profile. Therefore, both Glucomannan and Gelatin are good options to be used as a gelling agent in the gummy base due to the excellent drug release profile obtained.

#### 3.1.6. Stability Studies

Table 7 displays the findings from the accelerated stability experiments of F1 and F2. The physical state of the gummies, pH, viscosity, and assay showed no significant difference between the initial and final samples (*p* > 0.05). Depending on the study findings, samples might be kept in amber glass bottles or airtight HDPE containers. The final formulas’ viscosity data, like the original formulas, also suggested stability over a more extended period.

**Figure 4:**
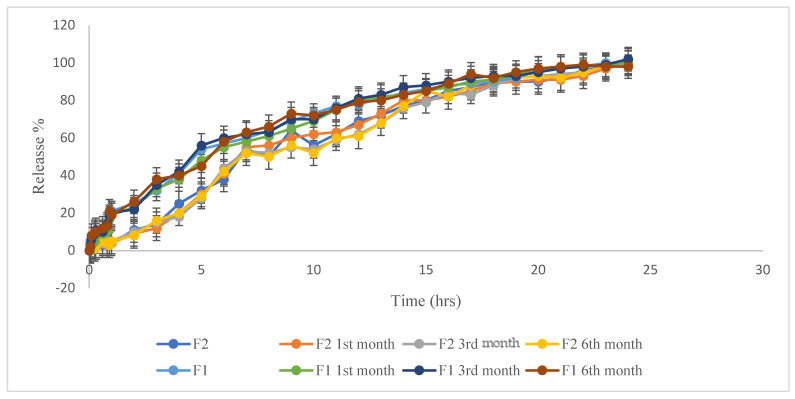
The release profile for F1, and F2 after, 1st, 3rd, and 6th month of stability testing at 5 ± 3 °C at pH 1.2 in the 1st 2 h and up to 24 h at pH 6.8.

The dissolution profiles of the produced formulas in the first 2 h at pH 1.2 and up to 24 h at pH 6.8 for F1 and F2 after the 1st, 3rd, and 6th month of stability testing at 5 ± 3 °C are shown in Figure 4. From the release profile, F1 and F2 gave almost the same release profile after the 1st, 3rd, and 6th month of stability testing, which is almost superimposable with the freshly prepared formulas. A full release of Risperidone was obtained as well. So, we can conclude that F1 and F2 are stable at 5 ± 3 °C.

### 3.2. Drug Characterization

#### 3.2.1. Differential Scanning Calorimetry (DSC)

**Figure 5:**
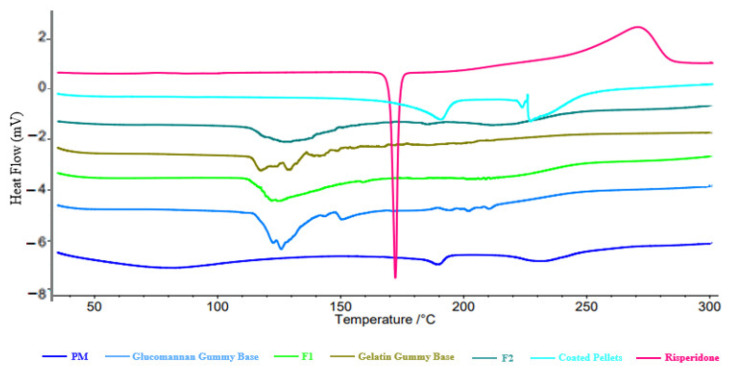
DSC thermogram for PM, Glucomannan Gummy Base, F1, Gelatin Gummy Base, F2, Coated Pellets, and Risperidone.

As shown in Figure 5, a sharp peak for Risperidone at 172.2 °C is similar to that as reported in the literature. This indicates the purity of the drug, which indicates the melting temperature of Risperidone, which ranges from 170.5 to 175.3 °C [55]. Risperidone did not exhibit any typical peaks in the coated pellets, indicating that the drug became more amorphous during the encapsulation process. This peak was presented in the PM with a lower intensity and a bit of shifting due to the dilution effect of other excipients. In the case of the drug-loaded gummy base, melting point determination of Risperidone was not easy, which suggests that a solid solution had been developed composed of the drug and the excipients used in the gummy, and this solid solution is present in an amorphous state in both formulas F1 and F2. In the case of F1 and F2, it was noticed that only a broad endothermic transition was found between 120–160 °C for F1 and between 125–180 °C for F2, conforming moisture loss due to heating from the gummy base.

#### 3.2.2. X-ray diffraction (XRD)

**Figure 6:**
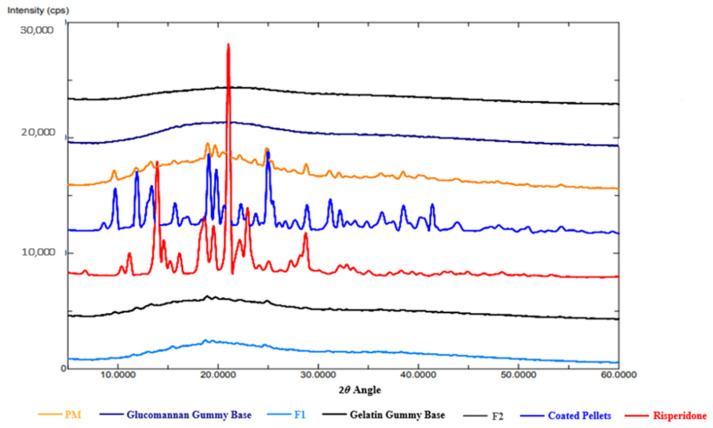
XRD patterns for PM, Glucomannan Gummy Base, F1, Gelatin Gummy Base, F2, Coated Pellets, and Risperidone.

XRD analysis shown in Figure 6 confirmed the previously stated DSC results. Where, Risperidone diffractogram exhibited a characteristic peak at 14°, 15°, 19°, 21°, 22°, 23°, 24°, 33°, and 34° indicating a high degree of crystallinity of the pure Risperidone. But in the case of F1 and F2 formulation, it was noticed that there was a total disappearance of the sharp peaks of Risperidone in the diffractogram, suggesting that Risperidone was in an amorphous state [56].

## 4. Conclusions

This study aimed to develop a medicated gummy dosage form for autistic children diagnosed with ADHD. It involves making chewable gummies with coated Risperidone pellets. These gummies, called F1 and F2, had pH values near the acceptable acidic pH values for gummy formations. Both F1 and F2 showed a non-Newtonian pseudo-plastic flow characteristic (shear-thinning), where the viscosity of F2 was higher than F1. Both of the formulas met the USP standards for weight uniformity and drug content. Relaying on the drug release profiles for F1, F1 crushed, F2, and F2 crushed, both Glucomannan and Gelatin are good candidates for use as a gelling agent in the gummy base due to their effect on delaying the drug release, and preventing burst drug release. Depending on the stability data, the produced formulas were found to be stable for 6 months at 5 ± 3 °C. So, it is noteworthy that this research opens up new possibilities for mixing modern and traditional medicine production methods.

Future work is needed to evaluate formula pharmacokinetics in animals and the pharmacodynamics using this formula with an ADHD-induced animal model to assess the effect of using Pycnogenol^®^ in the formula on ADHD symptoms, as well as to evaluate how the use of Glucomannan influences weight gain, which represents the main side effect of Risperidone. Also, an accelerated stability study at 25 °C ± 2 °C/60% RH ± 5% RH for 6 months must be conducted.

## Figures and Tables

**Table 1:** The fluid-bed granulator parameters used during the preparation of coated Risperidone pellets.

Working Parameters	Values
Inlet air temperature	50–55 °C
Exhaust air temperature	30–50 °C
Product temperature	40–43 °C
Coating dispersion temperature	Room temperature
Injection rate *	1, 2, 3, 4, 5, up to 10 RPM
Spraying air	0.8 bar
Flap	Starting from 30–50 bar
Shaking time	5–8 s
Pause time	2 min

* Note: injection rate increases at 15 min intervals.

**Table 2:** Ingredients used in gummies formulation.

Ingredients	F1	F2
Solution (A)		
Sucrose	30%	30%
Glucose D60	60%	60%
Water	10%	10%
Solution (B)		
Glucomannan	30%	
Gelatin		30%
Water	70%	70%
Rest of ingredients		
Potassium sorbate	8 mL	8 mL
Citric acid	25% of saccharose quantity	25% of saccharose quantity
Strawberry concentrate	0.5%	0.5%
Dye (red color)	0.5% weight of water	0.5% weight of water

**Table 3:** Apparatus used, reagents needed, and the procedure used in the determination of Risperidone content.

Apparatus Used	HPLC-UV
Reagents	Methanol HPLC grade, Purified water, and buffer (5 g Ammonium acetate in 1000 mL water) (dissolve 5 g Ammonium acetate in 1000 mL water).
Procedure	
Column used	Hypersil BDS, C18 (250 × 4.6) mm, 5 µm.
Mobile phase	Buffer solution: Methanol (450/550)
Flow rate	1.0 mL/min
Wavelength	276 nm
Retention Time	About 11 min
Injection volume	20 µL
Diluent	Mobile phase

**Table 4:** Apparatus used, reagents needed, and the procedure for in vitro drug release.

Apparatus Used	HPLC-UV, Dissolution Tester (Apparatus II).
Reagents	Hydrochloric acid (37%) (AR grade), Acetonitrile (HPLC grade), trifluralin acid Ammonium hydroxide solution (25%), Purified water.
Dissolution conditions	
Medium	0.1 N HCL, Buffer solution pH 6.8
Medium volume	500 mL
apparatus	II (Paddle)
Number of RPM	50 RPM
Temperature	37.0 ± 0.5 °C
Time	2 h in 0.1 N HCL, and 24 h in Buffer solution PH 6.8
Chromatographic conditions	
Column used	Hypersil BDS, C18 (250 × 4.6) mm, 5 µm.
Mobile phase	The water and Acetonitrile mixture (65:35) was filtered and degassed 0 ml of Trifluoroacetic acid was then added to each 1 L mixture. The pH was adjusted to 3 by Ammonium hydroxide.
Auto-sampler temperature	10 °C
Column oven temperature	25 °C
Wavelength	237 nm
Flow rate	1.5 mL/min
Retention Time	About 2.7 min
Injection volume	50 µL
Diluent	0.1N HCL

**Table 5:** The pH value for formulas F1 and F2.

Formula No.	pH Value	±SD
F1	3.46	0.01
F2	3.42	0.02

**Table 6:** Formulas F1, and F2 weight uniformity.

Formula	Mold Type	Mean Weight (g) ± SD	Weight Variation % Limits
F1	Circular	2.882 ± 0.016	−0.99–0.54
	Heart shape	2.276 ± 0.013	−0.03–1.48
	Bear-shaped	1.901 ± 0.011	−0.61–0.74
F2	Circular	2.932 ± 0.018	−0.27–2.25
	Heart shape	2.465 ± 0.015	−0.46–2.11
	Bear-shaped	2.012 ± 0.014	−0.08–3.41

**Table 7:** Accelerated stability study results for formulas F1, and F2.

Time Duration	F1				F2			
	Organoleptic Characteristics	pH	Viscosity	Essay	Organoleptic Characteristics	pH	Viscosity	Essay
Initial	Initial Red color	3.46 ± 0.01	170 ± 11.05	102.4 ± 4.23%	Initial Red color	3.42 ± 0.02	1040 ± 34.6	99.96 ± 3.58%
15 days	No change in color	3.48 ± 0.04	170 ± 10.14	101.81 ± 1.34%	No change in color	3.42 ± 0.03	1039 ± 41.63	100.2 ± 1.52%
1 month	No change in color	3.46 ± 0.05	171 ± 8.44	103.05 ± 1.51%	No change in color	3.44 ± 0.07	1038 ± 38.82	99.99 ± 2.95%
3 months	No change in color	3.51 ± 0.13	169 ± 8.97	100.66 ± 6.22%	No change in color	3.48 ± 0.04	1041 ± 43.05	101.04 ± 0.71%
6 months	No change in color	3.45 ± 0.08	170 ± 12.53	101.64 ± 0.97%	No change in color	3.45 ± 0.01	1040 ± 30.97	99.93 ± 2.23%

## Data Availability

Data is contained within the article.
